# Percutaneous Cryoablation of Central Liver Tumors with Thermal Protection of the Bile Duct

**DOI:** 10.1007/s00270-025-04146-z

**Published:** 2025-08-12

**Authors:** Daniel Markus Düx, Benjamin Heidrich, Frank Wacker, Kristina Imeen Ringe

**Affiliations:** 1 Department of Diagnostic and Interventional Radiology, Hannover Medical School, Hannover, Germany; 2 Department of Gastroenterology, Hepatology Infectious Diseases and Endocrinology, Hannover Medical School, Hannover, Germany

**Keywords:** Cryoablation, Hepatocellular carcinoma, Liver metastases, Bile ducts

## Abstract

**Purpose:**

To assess the technical feasibility and outcome of cryoablation with simultaneous bile duct protection in patients with centrally located liver tumors (CLLT).

**Material and Methods:**

Three patients (all male, 40–79 years) with CLLT located in close proximity to the right (*n* = 2) or left (*n* = 1) hepatic duct were included in this retrospective study. Prior to cryoablation, a nasobiliary tube was placed endoscopically in the respective bile duct. In two cases, additional temporary bile duct stent placement was performed. CT-guided cryoablation was realized with simultaneous bile duct perfusion using warm saline. Technical success, effectivity and complications were assessed.

**Results:**

Three CLLT (hepatocellular carcinoma (HCC) *n* = 2; metastasis *n* = 1; tumor size 9–15 mm) were treated. Cryoablation was technically successful in all cases and treatment effectivity (A0 ablation) was 100%. Nasobiliary tubes were removed immediately after the ablation procedure, bile duct stents were removed –six to eight weeks after ablation. At the latest available follow-up for each patient (909, 343 and 32 days), no bile duct-related complications were observed. No local tumor recurrence was observed in the two patients who survived longer than three months.

**Conclusion:**

According to our initial experience, percutaneous cryoablation of CLLT with simultaneous bile duct protection is technically feasible without an increased risk of biliary complications and local tumor recurrence.

**Graphical Abstract:**

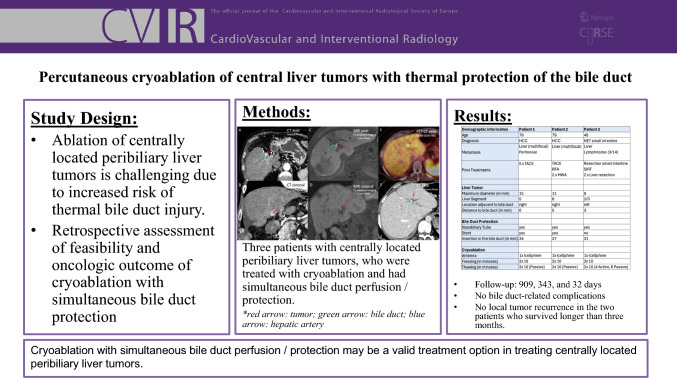

## Introduction

Image-guided percutaneous tumor ablation is a well-established minimally invasive procedure for treating liver tumors, offering excellent outcomes and becoming a crucial tool in interdisciplinary patient management. Heat-based techniques, including radiofrequency ablation (RFA) and microwave ablation (MWA), are incorporated in international guidelines for liver tumor treatment [1].

Ablation of centrally located liver tumors (CLLT) remains challenging due to increased risk of thermal injury to sensitive structures. To minimize biliary complications, tumors within 1 cm of the main biliary tract should not be treated unless protective measures like active bile duct cooling are performed [2]. This involves infusing a 5% dextrose-water solution or saline into the proximal bile ducts near the target lesion through an endoscopically placed nasobiliary tube or a percutaneous transhepatic biliary catheter before ablation [3]. Experience with these thermo-protective measures is limited to heat-based ablation techniques, with complication rates related to biliary injury ranging from 0 to 18% [4–6].

Cryoablation is currently experiencing a revival with some potential advantages compared to RFA or MWA, such as enhanced treatment monitoring with clear visualization of the ice ball formation and preservation of tissue matrix for healing, making it less damaging in cases of unintended nontarget ablation. While safety and efficacy of cryoablation for liver tumors has been assessed, evidence for treating CLLT is limited [7–11]. The incidence and severity of biliary complications are significantly lower with cryoablation than with RFA (28% vs. 68%; *p* = 0.007) when treating periductal liver tumors [7], and local control tumor control is similar to RFA or MWA [8].

Bile duct protection during cryoablation of CLLT has been described in one patient establishing a percutaneous transhepatic biliary drainage (PTCD) [12]. We share our initial experience in treating CLLT with cryoablation and simultaneous bile duct protection trough a nasobiliary tube.

## Material and Methods

This IRB-approved observational study identified all patients from our institutional database, who underwent percutaneous cryoablation of CLLT with simultaneous biliary duct protection. All patients required special consideration due to an increased risk of bile duct injury, and were selected for cryoablation with bile duct protection in our multidisciplinary tumor board.

### Preparation

Before cryoablation, an 8.5 French nasobiliary tube was inserted endoscopically in the bile duct near the target lesion. The tube was positioned in the right hepatic duct (*n* = 2) and in the left hepatic duct (*n* = 1). Antibiotics were applied during the procedure. Additionally, temporary bile duct stents were placed in two cases, while stent placement was technically not feasible in the third case. The rationale for stent placement was to ensure the bile duct’s patency, (1) to prevent acute bile duct obstruction from swelling after ablation, and (2) secondary bile duct obstruction from scarring. No further scarring is expected to occur six weeks after the ablation.

### Cryoablation

All ablations were performed in general anesthesia, under CT-guidance (Somatom xcite, Siemens Healthineers), using the VisualIce system (Boston Scientific), by one radiologist (> 15 years of experience in percutaneous ablations). Continuous bile duct perfusion with warm saline (≈ 40–45 °C) at a rate of 1–2 ml/s was maintained through the nasobiliary tube during the procedure. Ablation data are summarized in Table 1.

**Table 1:** Demographic information, tumor characteristics and technical data

Demographic information	Patient 1	Patient 2	Patient 3
Age	79	79	40
Diagnosis	HCC	HCC	NET small intestine
Metastasis	liver (multifocal) peritoneal	liver (multifocal)	liver lymphnodes (3/14)
Prior treatments	5 × TACE	TACE RFA 2 × MWA	Resection small intestine SIRT 2 × liver resection
Liver tumor			
Maximum diameter (in mm)	15	11	9
Liver segment	5	8	2/3
Location adjacent to bile duct	right	right	left
Distance to bile duct (in mm)	0	5	3
Bile duct protection			
Nasobiliary tube	yes	yes	yes
Stent	yes	yes	no
Insertion in the bile duct (in mm)	34	37	31
Cryoablation			
Antenna	l × Ice sphere	l × Ice sphere	l × Ice sphere
Freezing (in minutes)	2 × 10	2 × 10	2 × 10
Thawing (in minutes)	2 × 10 (passive)	2 × 10 (passive)	2 × 10 (4 active, 6 passive)

### Follow-up

Nasobiliary tubes were removed the same day, bile stents six weeks later. Complications were assessed according to the CIRSE Classification System [13]. Last available follow-up was used to report local tumor recurrence (LTR), defined as recurrent tumor within or immediately adjacent to the ablation zone, and disease progression [14].

## Results

**Fig. 1:**
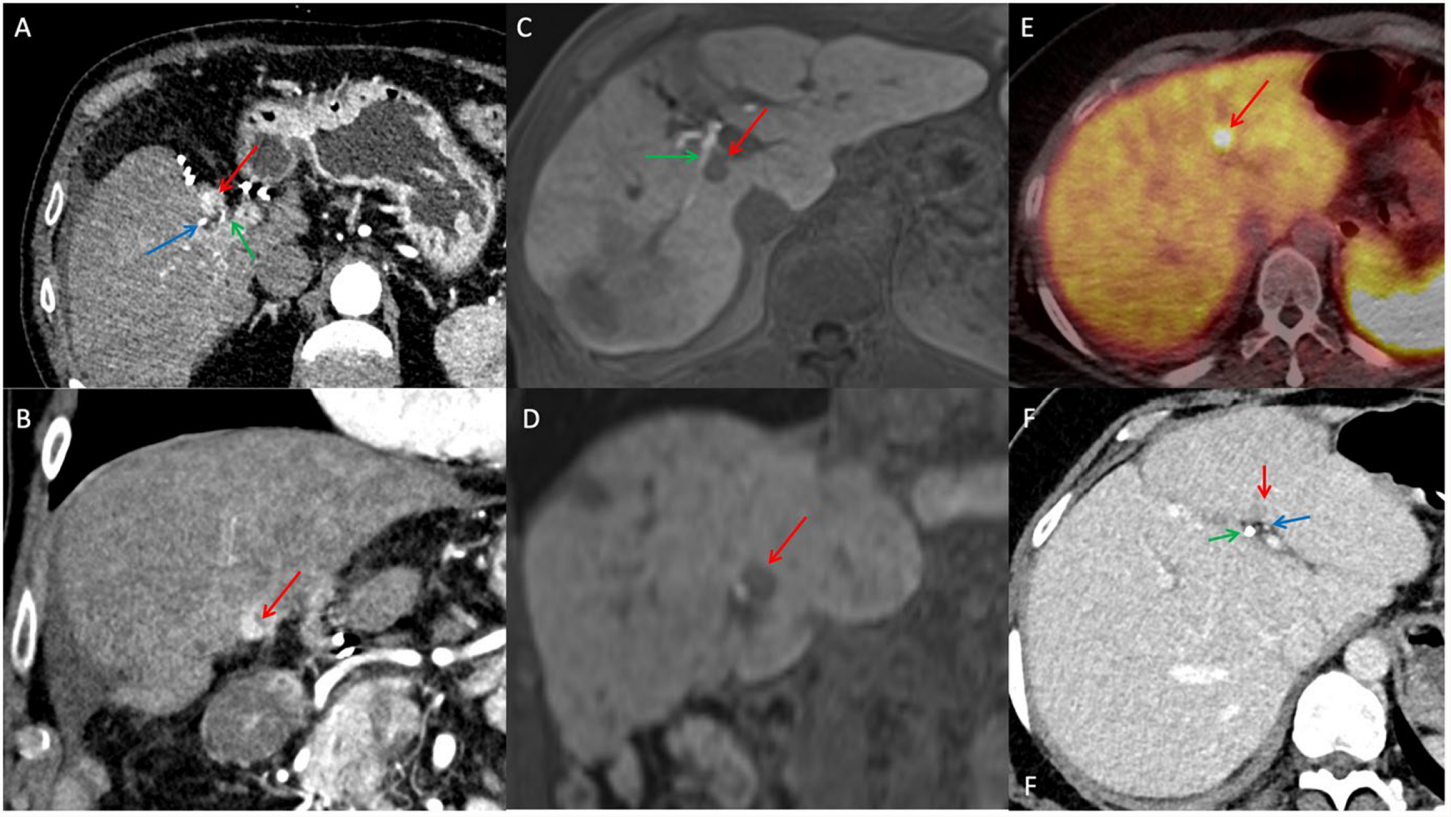
All patients with centrally located liver cancer with pre-interventional imaging before CT-guided cryoablation. Three patients with CLTT are presented: Patient 1 and Patient 2 (**A–D**) both have HCC adjacent to the right hepatic duct, while Patient 3 (**E, F**) has a hepatic metastasis of a NET near the left hepatic duct. All patients required extra consideration with protective bile duct measures to minimize the risk of bile duct injury associated with ablation in this central tumor location. Our interdisciplinary tumor board recommended CT-guided cryoablation with bile duct protection via nasobiliary drainage, enabling the perfusion of bile ducts with warm saline during the ablation procedure. *red arrow: tumor; green arrow: bile duct; blue arrow: hepatic artery. *HCC: Hepatocellular carcinoma; NET: neuroendocrine tumor. **A + B:** CT in axial and coronal orientation (arterial phase). **C + D:** T1-weighted MRI in axial and coronal orientation (delayed phase). **E:** PET-CT in axial orientation (Ga-68-DOTA-TATE). **F:** CT in axial orientation (portal venous phase)

Three patients were eligible for cryoablation of CLLT (hepatocellular carcinoma (HCC) *n* = 2; metastasis *n* = 1; tumor size 9–15 mm) with simultaneous bile duct protection between 10/18 and 08/23 (Fig. 1). Demographic information, tumor characteristics and technical data are summarized in Table 1.

Patient 1 had multifocal HCC classified as Barcelona Clinic Liver Cancer classification (BCLC) stage C with two peritoneal metastases, which were successfully resected four years ago. Since then, no extrahepatic metastasis occurred. Although TACE was the preferred treatment for this tumor, an intrahepatic shunt made a 6th TACE procedure unfeasible, and CT-guided cryoablation was done for local tumor control.

Patient 2 had an HCC BCLC stage B and initially received TACE and RFA, remaining disease-free for four years. He was then successfully treated with MWA. Due to a single recurrence of CLLT, he underwent CT-guided cryoablation 21 months after the MWA.

Patient 3 had a neuroendocrine tumor (NET) 20 months prior to the cryoablation. Primary oncologic resection was performed to address the tumor and locoregional lymph nodes, followed by selective internal radiotherapy (SIRT) to treat metastatic disease in liver segment 4. All liver metastases were surgically removed except for one close to left proximal bile duct that was not amenable for operation due to its central location.

**Fig. 2:**
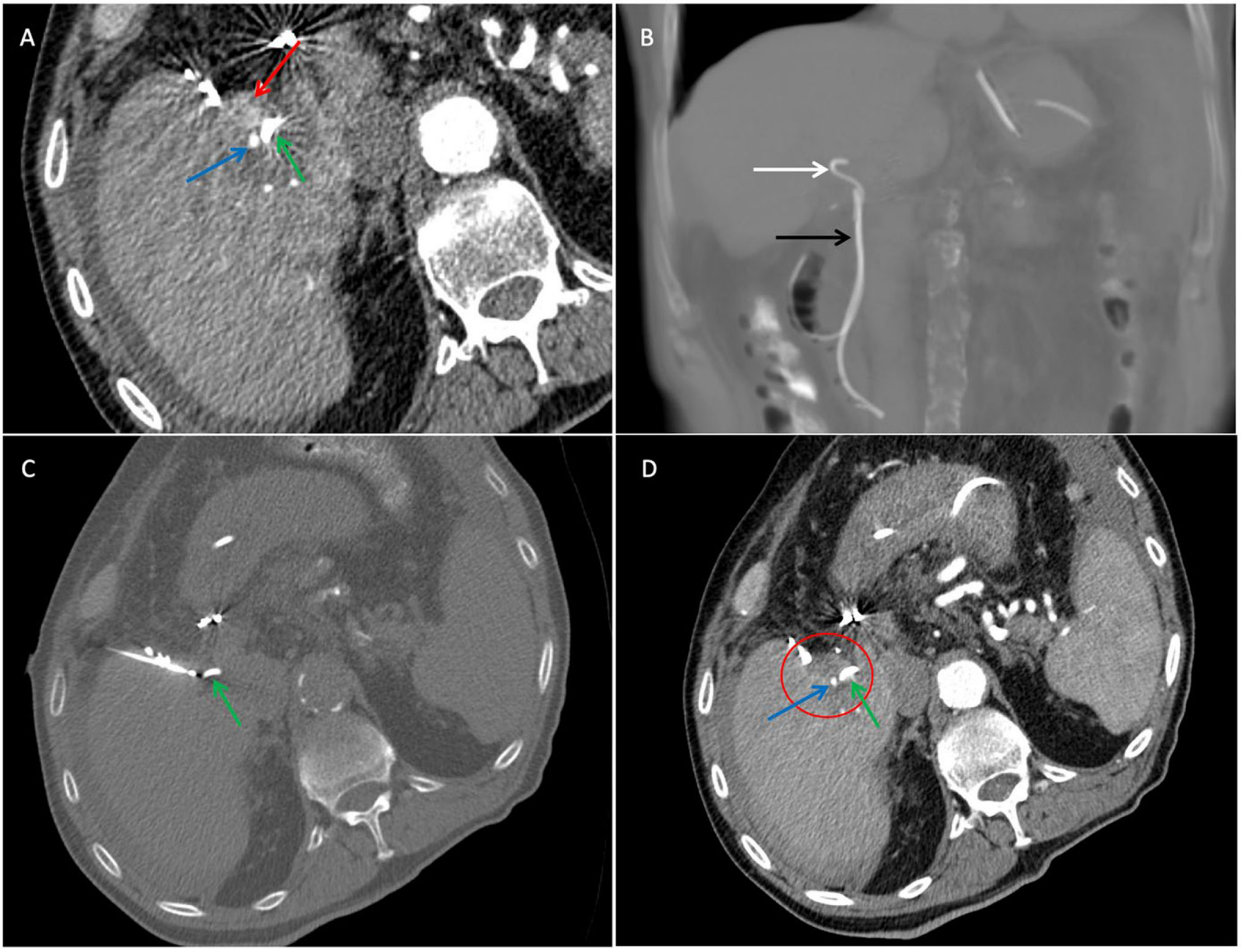
CT-guided cryoablation of patient 1 with HCC next to the right hepatic duct. **A:** Pre-ablation planning CT (axial in arterial phase) shows a 15-mm HCC with early contrast enhancement next to the right hepatic duct and hepatic artery. The minimum distance to the right hepatic duct was 0 mm. **B:** Pre-ablation planning CT (coronal without contrast medium) shows the nasobiliary tube and biliary stent, which was placed 34 mm into the right hepatic duct before cryoablation. **C:** Position of the cryoprobe just before ablation (CT axial without contrast medium). **D:** Post-ablation CT (axial in arterial phase) shows complete necrosis of the HCC with inflammatory rim enhancement.*red arrow: tumor; green arrow: bile duct; blue arrow: hepatic artery; black arrow: nasobiliary tube; white arrow: biliary stent. *HCC: Hepatocellular carcinoma

Preoperative endoscopically placement of a nasobiliary drainage was performed in all patients without complications (Fig. 2). Cryoablation was technically successful without complications in all patients and treatment effectivity (A0 ablation) was 100% in immediate post-ablation CT.

**Fig. 3:**
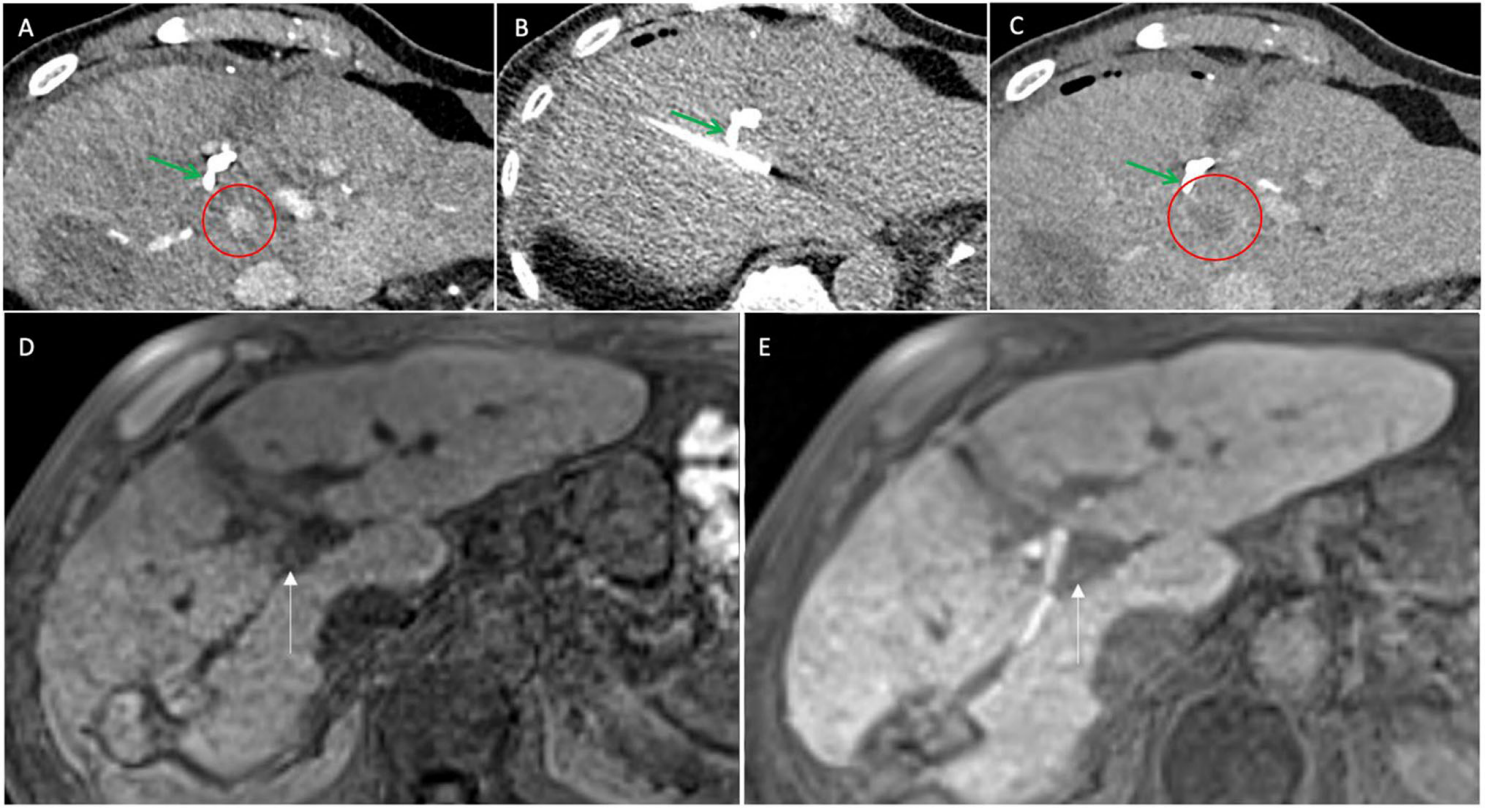
CT-guided cryoablation of patient 2 with HCC near the right hepatic duct and 30 months follow-up MRI. **A:** Pre-ablation planning CT (axial in portal venous phase) shows a 11-mm HCC with early contrast enhancement next to the right hepatic duct with a minimum distance of 5 mm. **B:** Position of the cryoprobe just before ablation (CT axial without contrast medium). **C:** Post-ablation CT (axial in portal venous phase) shows complete necrosis of the HCC with inflammatory rim enhancement. **D, E:** Small focus of hemorrhage with settle hyperintensity on pre- and post-contrast T1-weighted MRI (axial), but no enhancement 30 months post-ablation, indicating no residual or recurrent HCC. Previous follow-up (not shown) revealed a larger area of hemorrhage that decreased over time. *red circle: tumor/post-ablation necrosis; green arrow: bile duct; white arrow: follow-up area at the site of the previous HCC. *HCC: Hepatocellular carcinoma

**Fig. 4:**
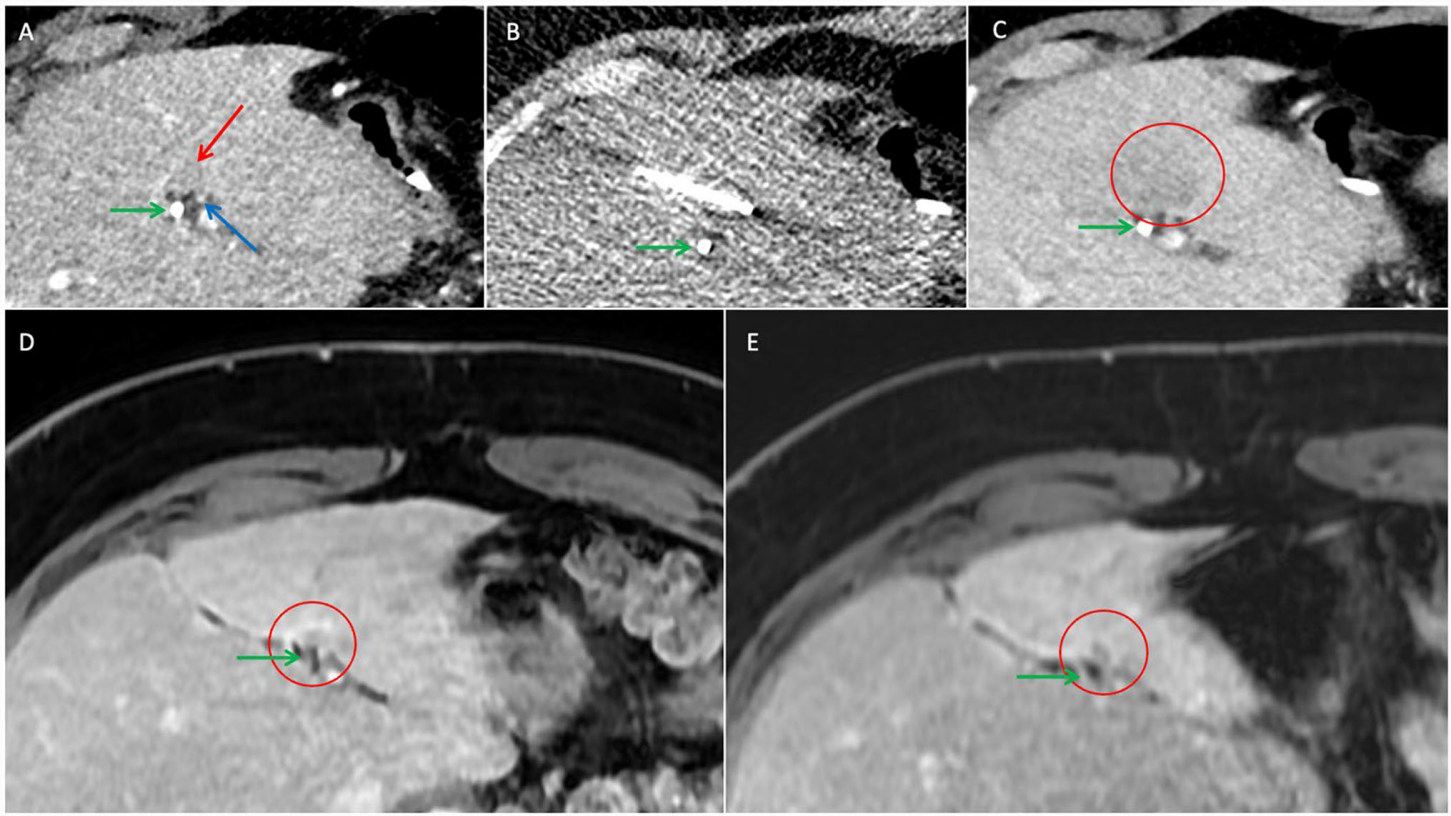
CT-guided cryoablation of patient 3 with a NET metastasis near the left hepatic duct and follow-up (2 and 11 months) CTs. **A:** Pre-ablation planning CT shows a 9-mm hypodense NET next to the left hepatic duct with a minimum distance of 3 mm. **B:** Position of the cryoprobe just before ablation. **C:** Post-ablation imaging shows complete necrosis of the HCC with inflammatory rim enhancement. **D, E:** The ablation zone continuously is shrinking with reduced inflammatory rim enhancement at the 2- and 11-month follow-up, indicating no residual or recurrent tumor. *red arrow: tumor; *red circle: post-ablation necrosis; green arrow: bile duct; blue arrow: hepatic artery. *NET: Neuroendocrine tumor

At the latest available follow-up for each patient (909, 343, and 32 days), no bile duct-related complications were observed. No LTR was observed in the two patients who survived longer than three months. One patient (Fig. 2) died 32 days post-ablation from a nontreatment-related cause. Another patient with HCC developed two new metastases in liver segments 7 and 8 after stopping Atezolizumab and Bevacizumab due to myositis 12 months post-ablation, which were resected. This patient remained tumor-free 909 days post-ablation, with no residual or recurrent tumor at the treatment site (Fig. 3). The patient with metastasized NET maintained stable disease and showed no LTR 343 days post-ablation (Fig. 4).

## Discussion

Ablating centrally located liver tumors (CLLT) presents significant challenges due to their proximity to major bile ducts, which can cause severe complications [15]. The novelty of our study is the assessment of safety and efficacy of cryoablation in CLLT with simultaneously warming the bile ducts trough a nasobiliary tube to enhance the cold sink effect for bile duct protection [16]. A similar technique has been described for cryoablation through a PTCD in one patient [12]. However, PTCD is more invasive than a nasobiliary tube and carries a higher risk of cholangitis. Additionally, it can be challenging to establish PTCD in patients without bile duct dilation. While this study is limited to technical aspects only, our study assessed oncologic outcomes demonstrating no local tumor recurrences in two patients who survived beyond three months (909 and 343 days).

Oncologic outcomes of heat ablation with simultaneous thermo-protective measures have been described [4–6]. The success of ablation of CLLT with thermo-protective measures is based on the sensitivity of the ablation method to bile duct perfusion. Increased sensitivity enhances the safety of the bile ducts but also raises the risk of residual disease due to incomplete ablation. Heat ablation poses significant challenges with LTR rates of 8–21%, which may be associated with the relatively high sensitivity of heat to the heat-sink effect [4, 5]. Our cryoablation approach theoretically reduces LTR risk due to its lower sensitivity to the cold sink effect, though it may pose a higher risk to bile ducts, which was not observed in our cohort [17, 18]. Cryoablation appears to balance sensitivity to the cold sink effect better than heat-based techniques. However, direct comparisons between ablation techniques in CLLT patients are missing and could provide further insights into cryoablation’s role in liver interventions. Additionally, the treatment of larger CLLT could be a valuable focus for future research.

Alternative promising and safe treatment options for CLLT are brachytherapy and laser-induced interstitial thermotherapy (LIIT). Brachytherapy achieved a 71.5% local-relapse-free survival rate at 12 months and LIIT showing a 74.1% initial efficacy rate with a 22% local tumor progression rate during a median follow-up of 20 months [19, 20]. However, both methods are limited to few centers, and the experience with LIIT’s is limited to few patient data.

## Conclusion

Cryoablation with simultaneous bile duct perfusion/protection may be a valid treatment option in treating CLLT. The small size and the lack of follow-up for one patient limit the generalizability of the efficacy and safety of this study

## Abbreviations

BCLC: Barcelona clinic liver cancer classification
CLLT: Centrally located liver tumors
CT: Computed tomography
HCC: Hepatocellular carcinoma
IRB: Institutional review board
LTR: Local tumor recurrence
LIIT: Laser-induced interstitial thermotherapy
MRI: Magnetic resonance imaging
MWA: Microwave ablation
MASH: Metabolic dysfunction-associated steatohepatitis
PTCD: Percutaneous transhepatic cholangiography and drainage
RFA: Radiofrequency ablation
SIRT: Selective internal radiotherapy
TACE: Transarterial chemoembolization
